# Preparation and use of *Xenopus* egg extracts to study DNA replication and chromatin associated proteins

**DOI:** 10.1016/j.ymeth.2012.03.029

**Published:** 2012-06

**Authors:** Peter J. Gillespie, Agnieszka Gambus, J. Julian Blow

**Affiliations:** a Wellcome Trust Centre for Gene Regulation & Expression, University of Dundee, Dow Street, Dundee DD1 5EH, UK; b School of Cancer Sciences, University of Birmingham, Edgbaston, Birmingham B15 2TT, UK

**Keywords:** *Xenopus*, Egg extract, *In vitro*, Cell-free system, DNA replication, Chromatin

## Abstract

The use of cell-free extracts prepared from eggs of the South African clawed toad, *Xenopus laevis*, has led to many important discoveries in cell cycle research. These egg extracts recapitulate the key nuclear transitions of the eukaryotic cell cycle *in vitro* under apparently the same controls that exist *in vivo*. DNA added to the extract is first assembled into a nucleus and is then efficiently replicated. Progression of the extract into mitosis then allows the separation of paired sister chromatids. The *Xenopus* cell-free system is therefore uniquely suited to the study of the mechanisms, dynamics and integration of cell cycle regulated processes at a biochemical level. In this article we describe methods currently in use in our laboratory for the preparation of *Xenopus* egg extracts and demembranated sperm nuclei for the study of DNA replication *in vitro*. We also detail how DNA replication can be quantified in this system. In addition, we describe methods for isolating chromatin and chromatin-bound protein complexes from egg extracts. These recently developed and revised techniques provide a practical starting point for investigating the function of proteins involved in DNA replication.

## 1 Introduction

Ever since their development and first use almost 30 years ago, cell-free extracts prepared from frogs’ eggs have enabled key discoveries in cell cycle research [1,2]. Ongoing research in fields as diverse as the control of cell cycle progression, apoptosis, nuclear formation, nucleocytoplasmic transport, spindle microtubule dynamics, chromatin structure, sister chromatid cohesion and the regulation of DNA replication, prove the continuing value of this established model system.

In the absence of an intact cell, these soluble egg extracts recapitulate the key nuclear transitions of the eukaryotic cell cycle *in vitro* under apparently the same controls that exist *in vivo*. DNA added to the extracts is first assembled into chromatin and then into structures corresponding to interphase nuclei. Once nuclear assembly is complete, the DNA is efficiently duplicated, producing a full complement of paired sister chromatids. Advancement of the extracts into mitosis supports first the condensation and resolution of the paired sisters and ultimately their separation on the spindle, all *in vitro*. That these extracts support this wide range of activities *in vitro* makes this model uniquely suited to the study of the mechanisms, dynamics and integration of cell cycle regulated processes at a biochemical level.

The ability of *Xenopus* egg extracts to support cell cycle progression *in vitro* depends on some key properties of early *Xenopus* embryos. As in other vertebrates, *Xenopus* eggs are arrested in metaphase of meiosis II. Fertilization promotes completion of meiosis, the extrusion of a polar body and progression into interphase of the mitotic cell cycle. In only 7 h after fertilization they undergo 11 synchronous rounds of cell division in the absence of significant transcription; only after this stage (the ‘Mid-Blastula Transition’) does zygotic transcription occur [3]. Most of the proteins required for cell cycle progression are pre-formed in the egg, and the continuing translation of a single protein (cyclin B) can support passage through the whole cell cycle [4]. For this reason, *Xenopus* egg extracts contain abundant stockpiles of material to support the nuclear assembly and replication of DNA up to concentrations typically seen at the onset of zygotic transcription (cell cycle 12, with ∼4000 cells per embryo) [5].

The regulation of DNA replication in *Xenopus* egg extract occurs under the same cell cycle controls as occur *in vivo* [5–7]. The complete duplication of chromosomal DNA in the *Xenopus* cell-free system requires the activation of many thousands of replication origins. To ensure genetic stability these origins must fire once and only once in each cell cycle. To this end two distinct signals are required to permit origin activation [8–10]. During late mitosis, and early G1, replication origins are first ‘licensed’ for subsequent DNA replication by being loaded with double hexamers of the Mcm2-7 proteins [11–13]. During S-phase, replication forks are initiated at licensed origins by the combined action of two protein kinases: S phase CDKs and Dbf4-dependent kinases (DDKs). Before these kinases can promote initiation of replication forks, the DNA must be assembled into an intact interphase nucleus; nuclear protein import also prevents any further origins from being licensed by activating the licensing inhibitor geminin [7,8,14–17]. The combination of CDK and DDK activity promotes the recruitment of replisome proteins to origins which activates the helicase activity of Mcm2-7 to unwind template DNA at the replication fork. Origins therefore revert to an unlicensed state upon initiation as Mcm2-7 moves away with the replication forks. The sequential activation of the license and initiation signals ensures faithful duplication of the genome.

In addition to permitting replication of the genome, origin licensing coordinates other cell cycle activities. The chromatin association of Mcm2-7 both coordinates replication licensing with cohesin chromatin association [18,19] and facilitates nuclear assembly [20]. In addition, changes in the dynamic association of a range of other nuclear proteins with chromatin is seen in response to licensing [21]. Replication licensing therefore serves as a major integration point of the early cell cycle. This integration serves to ensure rapid passage through the first cell cycles during *Xenopus* early development.

In this article we describe the methods currently in use in our laboratory for the study of DNA replication using the *Xenopus* cell-free system. In addition to a description of the methods used for extract preparation, we detail their application for studying the dynamic association of proteins with chromatin. These recently developed and revised techniques provide a practical starting point for investigation of protein function using this system.

## 2 Preparation of *Xenopus laevis* egg extracts and demembranated sperm nuclei

### 2.1 Preparation of *X. laevis* egg extracts

From the original method of Lohka and Masui [1], small but significant optimisations have been developed for preparing *Xenopus* egg extracts suited for studying different aspects of the cell cycle. In particular, methods for preparing extracts that support efficient DNA replication differ from methods for preparing extracts that support efficient passage through mitosis. It should be noted that there is also a specialised method for preparing ‘nucleoplasmic’ extracts which can support the replication of DNA in the absence of nuclear assembly [22].

#### 2.1.1 General considerations for egg production

*Xenopus* eggs are arrested at metaphase of meiosis II. Addition of exogenous Ca^2+^, which mimics the calcium wave generated at fertilisation, activates these unfertilised eggs and extracts prepared from them, to enter the first mitotic interphase. Extracts prepared from metaphase arrested eggs in the presence of a Ca^2+^ chelator, such as EGTA, maintain the arrest [2]. The release of extracts from metaphase arrest activates the replication licensing system enabling the replication of exogenous DNA [9]. It is also possible to prepare extracts from eggs that have been briefly activated *in vivo* by treatment with a calcium ionophore. We typically find that extracts from activated eggs show a larger variability in quality than those prepared from unactivated eggs. However, ionophore activation is a useful technique when it is important to ensure exit from meiosis prior to extract preparation, for example if extracts that have efficiently activated the licensing system *in vivo* are required [11,23,24].

Eggs are first dejellied and then extracts are prepared by a series of centrifugation steps. Dejellied eggs are closely packed into centrifuge tubes by a very low speed spin which removes most of the wash buffer from the egg mass. The eggs are then spin-crushed, before a final clarifying spin yields an extract that can be frozen and stored under liquid nitrogen or at −80 °C. Stored extracts remain stable without loss of quality for a considerable period, at least 10 years in our experience. Whereas frozen extracts support efficient and rapid DNA replication, some other applications may demand the use of fresh extracts, in particular if extracts need to cycle in and out of mitosis *in vitro*. We have experience of using unfrozen extracts prepared by this procedure without concern.

The single most important consideration when preparing extracts is the quality of the starting material, the eggs. Eggs of the very best quality are essential for preparation of a high quality extract. In this regard it is vitally important that the *Xenopus* colony is maintained in prime health. To this end, care and handling of the frogs within the framework of local legislation and internationally accepted best practice guidelines are advised.

In order to increase the number of stage 6 mature oocytes female frogs are primed with 50 units of Folligon (Pregnant Mare Serum Gonadotrophin; Intervet) 3 days before the eggs are required. We typically inject ∼15 frogs per preparation. At approximately 4 pm on the day before eggs are required, frogs are injected with 500 units of Chorulon (Chorionic Gonadotrophin; Intervet) and placed in individual laying tanks at 19–23 °C in 2 l of laying buffer. To ensure that only the freshest eggs are used for extract preparation the first collection of the day is made by 9.30 am. To avoid unnecessary delay and ensure swift extract preparation all materials required during the process are readied prior to beginning: solutions are prepared, frozen stocks are thawed and stored appropriately and a selection of tubes are placed covered on ice.

**Fig. 1:**
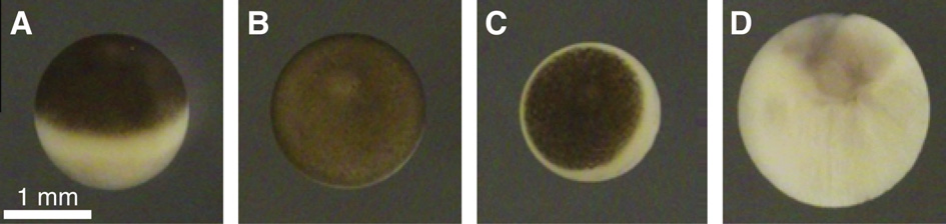
*Xenopus* eggs. (A and B) Unactivated, meiosis II metaphase arrested eggs, (C) activated egg and (D) apoptotic egg. A, C and D show top views; B shows side view.

Metaphase arrested eggs can be identified by the clear circumferential distinction between the larger sized dark coloured animal pole and the smaller sized lightly coloured vegetal pole (Fig. 1A and B). The animal pole contracts upon activation, so that from the top eggs appear almost totally white but for a small black dot (Fig. 1C). When eggs spontaneously enter interphase in the absence of fertilization they often develop problems and apoptose in due course. Apoptotic eggs, which may float on the top surface of the egg mass, appear as large white or grey spheres, often visibly greater in volume than an intact egg (Fig. 1D). Activated or apoptotic eggs in a batch should be removed prior to spin crushing, using a Pasteur pipette or equivalent. Once crushed, the contents of the eggs mix together. Since activation and apoptosis are enzyme mediated processes, a small contamination with active or apoptotic eggs may render your extract useless. It is therefore advisable to remain vigilant and make considerable effort to remove activated and apoptotic eggs from your preparation during the early stages. The longer the time that the eggs lie before extract preparation the greater the likelihood there is of the eggs spontaneously activating and/or apoptosing. Collection of the eggs into a high salt buffer helps maintain the metaphase arrest. We find the egg collection buffer we currently use, MMR [25], is optimal in this regard. Furthermore, to guarantee egg freshness we make a number of egg collections throughout the day.

We collect the eggs from individual frogs in separate glass beakers. The collected eggs are assessed for quality and pooled accordingly into at least 3 groups. The highest quality eggs, those visibly in metaphase and containing no apoptotic or activated eggs, appear as a sea of black with the animal poles uppermost in a beaker of clear buffer. The best quality eggs remain visibly in metaphase during the early steps of extraction. In our experience eggs of the greatest dark/light contrast provide the best quality extracts. Extracts of pooled best quality eggs should be prepared and stored separately. The range in quality of eggs in the second class is wide, so a number of extracts may sensibly be prepared if egg volume allows. Preparing multiple extracts simultaneously, however, will slow preparation. A trade-off therefore has to be made between preparation time and the likely quality and volume of the final extract. Batches of eggs containing a small percentage that have either activated or apoptosed can be used for extract preparation after removing the undesirable eggs. Treat with caution any pool of eggs in which an increasing proportion are seen to activate or apoptose whilst on the bench before or during preparation. In this case, even though it may not yet be apparent visually, individual eggs may be in transition and in time a significant proportion will likely activate or apoptose. The inclusion of these eggs in an otherwise good preparation may render it useless. During laying the frogs may shed excessive amounts of skin or regurgitate food, discolouring the laying buffer. Extracts can be prepared from these ‘dirty’ eggs after removal of the detritus but we would not consider these eggs as high quality irrespective of their standard. On occasion a batch of eggs are so contaminated that they may be beyond sensible cleaning and these should be discarded. Other batches to be discarded are those in which a significant proportion have apoptosed and those in which long strings of apparently connected eggs are present. These long strings contain immature oocytes. These immature eggs are of an indeterminate cell cycle status. If considerable apoptosis has occurred the buffer may be turned from clear and colourless to opaque white-grey with the dispersed contents of the ruptured eggs. These eggs should be discarded.

We find that for best results, frogs should be given at least 4 months recovery time between induced ovulations. As the frogs age we find that both egg quality and quantity declines. We make considerable effort to keep detailed records of egg production. Typically, we find that frogs can yield 8–12 batches of eggs before egg quality or quantity become unacceptable.

#### 2.1.2 Materials and reagents

Suggested preparation day and storage temperature are abbreviated as follows:

−2D = Prepare 1 or 2 days prior to collection day.
IB = Prepare immediately before procedure.
RT = Room temperature.
RS = Refrigerated stock.
FS = Frozen stock.

*10× MMR (−2D, RT):* 1 M NaCl, 20 mM KCl, 10 mM MgCl_2_, 20 mM CaCl_2_, 1 mM EDTA, 50 mM HEPES–NaOH, pH 7.8.

*20× XB Salts (RS):* 2 M KCl, 40 mM MgCl_2_, 2 mM CaCl_2_.

*XBE2 (−2D, RS to RT):* 1× XB salts, 1.71% w:v sucrose, 5 mM K-EGTA, 10 mM HEPES–KOH, pH 7.7.

*LFB1/50 (FS to 4 °C):* 10% w:v sucrose, 50 mM KCl, 2 mM MgCl_2_, 1 mM EGTA, 2 mM DTT, 20 mM K_2_HPO_4_/KH_2_PO_4_ pH 8.0, 40 mM HEPES–KOH, pH 8.0.

*Energy regenerator (FS to 4 °C):* 1 M phosphocreatine K salt, 600 μg/ml creatine phosphokinase in 10 mM HEPES–KOH pH 7.6.

*Cytochalasin D (FS to RT):* 10 mg/ml in DMSO.

*Protease inhibitors (FS to RT):* aprotinin, 10 mg/ml in H_2_O; leupeptin, 10 mg/ml in H_2_O; pepstatin, 10 mg/ml in dimethylformamide.

*Dejellying solution (IB, RT):* 2% cysteine w:v in H_2_O, to pH 7.8 with NaOH.

#### 2.1.3 Egg extract preparation procedure

This protocol is based on one previously developed in our lab [23], with changes based on [26].

**Fig. 2:**
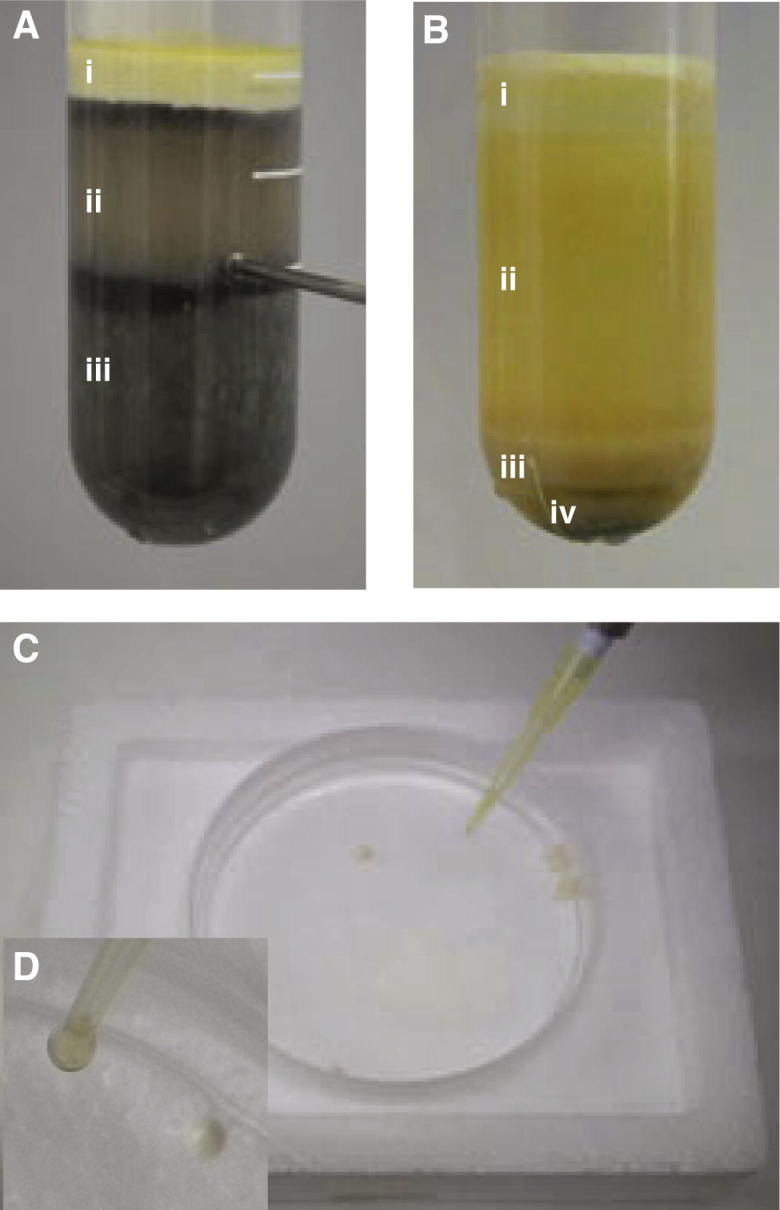
Preparation of *Xenopus* egg extracts. (A) Egg extract, post-crushing spin. (i) lipid layer; (ii) crude cytoplasm; (iii) yolk platelets. Recovery of the crude cytoplasm by side puncture is shown. (B) Egg extract, post-clarifying spin. (i) lipid layer; (ii) cytoplasm; (iii) membranous layer, contains mitochondria; (iv) residual yolk platelets and insoluble material. (C and D) Drop-freezing the final extract in 20 μl aliquots in liquid nitrogen using a cut pipette tip and a plastic Petri dish.

1. Egg volume and quality are recorded.
2. Rinse eggs several times in 1× MMR to remove non-egg debris.
3. Remove activated and apoptosed eggs with a Pasteur pipette.
4. After rinsing, pour off excess MMR and add dejellying solution. The solution is swirled gently to remove the jelly coat surrounding the egg. The dejellying solution may be removed and replaced several times to facilitate complete dejellying. When completely dejellied, the eggs pack tightly together.
5. Wash the dejellied eggs twice with room temperature XBE2 and then once with the XBE2 + protease inhibitors (final concentration 10 μg/ml for each component).
6. Transfer the eggs to 14 ml centrifuge tubes (containing 1 ml XBE2 + protease inhibitors + cytochalasin D (final concentration 100 μg/ml), using as few as possible. Remove excess buffer from the settling eggs.
7. Pack the eggs into the tube by centrifugation at 3000 rpm (∼1400*g*) in a Beckman JS13 (or similar) swinging bucket rotor for 1 min at 16 °C. Remove all excess buffer. Any remaining activated or apoptosed eggs resolve to the top surface during packing. Remove these eggs using a Pasteur pipette so that when looking down on the top of the tube the surface of the eggs appears black.
8. Spin-crush the eggs by centrifugation at 10,000 rpm (∼16,000*g*) in a Beckman JS13 (or similar) swinging bucket rotor for 10 min at 16 °C. The dirty brown cytoplasmic layer (approximately 1/3 of the sample, between the bright yellow lipid at the top and the grey yolk platelets at the bottom – layer II in Fig. 2A) is then collected using a 20G needle and a 1 ml syringe via side puncture. From this point onwards the extract is kept on ice.
9. Supplement the extract with a 1:1000 dilution of cytochalasin D (final concentration 10 μg/ml), 1:1000 dilution of protease inhibitors (final concentration of 10 μg/ml for each component), 1:80 dilution of energy regenerator and LFB1/50 to 15% v:v. Thoroughly mix the extract using a 3 ml plastic Pasteur pipette.
10. Load SW55 ultracentrifuge tubes (or equivalent) with 3–5 ml of extract on ice. Centrifuge the extract at 30,000 rpm (approximately 84,000*g*) in a pre-cooled SW55 rotor for 20 min at 4 °C. As shown in Fig. 2B, this spin results in a small black insoluble pellet (Fig. 2B-iv), a larger loose brown membranous pellet above it (Fig. 2B-iii), a clear golden cytoplasmic fraction (Fig. 2B-ii) with a variable amount of white membranous material floating in it, and a small yellow lipid plug (Fig. 2B-i).
11. Remove the lipid plug from the top of the tube with an ethanol-cleaned and dried small sized spatula. Then collect the golden cytoplasmic layer, including the wispy membranes floating immediately below the lipid plug. Do not disturb the ‘dirty yellow’ membrane layer below the cytoplasm (Fig. 2B-iii); this layer contains the mitochondria which promote apoptosis on freeze-thawing. If this layer is disturbed do not collect any more extract.
12. Supplement the recovered cytoplasm with 2% glycerol v:v and mix thoroughly but very gently using a 3 ml plastic Pasteur pipette. Record final extract volume.
13. Freeze the extract by dropping 20 μl aliquots into plastic Petri dishes containing liquid nitrogen (Fig. 2C) using a micropipette tip with the end cut off (Fig. 2D). The beads should then be stored under liquid nitrogen. Alternatively, place single use aliquots into appropriately sized Eppendorf tubes with needle-punctured caps, and freeze in liquid nitrogen; after freezing, the tubes may be stored at −80 °C.

### 2.2 Preparation of demembranated *X. laevis* sperm nuclei

*X. laevis* egg extracts are able to support the replication of a range of DNA templates including single and double stranded plasmids [5] and extracted mammalian nuclei [27]. The physiological substrate for use in the extracts is *X. laevis* sperm nuclei. The sperm nuclei, recovered from the testes post-mortem, remain stable for extended periods after demembranisation. This, in addition to the consistent and ready availability of these germ cells, makes demembranated sperm nuclei the preferred replicative substrate.

Sperm nuclei are highly condensed and individual chromosomes are held tightly packed together. This compaction is mediated by two highly basic sperm specific protamines that replace the bulk of histones H2A and H2B. When added to egg extract these protamines are removed and replaced by histones almost immediately in an exchange facilitated by nucleoplasmin [28]. The removal of the protamines promotes extensive decondensation of the sperm nuclei. This now remodelled chromatin serves as the substrate for the first mitotic interphase *in vitro*.

Demembranated sperm nuclei are prepared from isolated testes recovered post-mortem. We euthanise the frogs by means of a lethal dose of anaesthetic. Both induction of euthanasia and disposal of the cadavers are undertaken within the framework of local legislation. The preparation procedure is composed of two parts. In brief, after removing unwanted tissue from around the isolated testes the sperm are released from the lumen and seminiferous tissue by extensive chopping. The released nuclei are then demembranated and prepared for storage.

#### 2.2.1 Materials and reagents

Abbreviations as in Section 2.1.2.

*EB (−2D, RS):* 50 mM KCl, 5 mM MgCl_2_, 2 mM β-mercaptoethanol, 50 mM HEPES–KOH, pH 7.6.

*SuNaSp (IB to 4 °C):* 0.25 M sucrose, 75 mM NaCl, 0.5 mM spermidine, 0.15 mM spermine, 15 mM HEPES–KOH, pH 7.6.

*Lysolecithin (FS to 4 °C):* 5 mg/ml in H_2_O.

*Hoechst 33258 (FS):* 10 mg/ml in H_2_O; prepare 20 μg/ml dilution on the day.

*MS222 (IB, RT):* 0.2% w:v MS222, ∼0.5% w:v NaHCO_3_, to pH 7.5.

#### 2.2.2 Demembranated sperm nuclei preparation procedure

To increase sperm yield male frogs can be primed with 150 units Chorulon (Chorionic Gonadotrophin; Intervet) 5–9 days before the testes are removed. We typically prepare nuclei from 15 male frogs. The most time consuming steps of this procedure are the isolation of the testes and the recovery of the sperm immediately thereafter. The work can be divided up between a number of different people, some isolating testes and others recovering the sperm. All implements used during this procedure should be scrupulously clean. Ethanol rinse and dry already cleaned implements immediately prior to use.

**Fig. 3:**
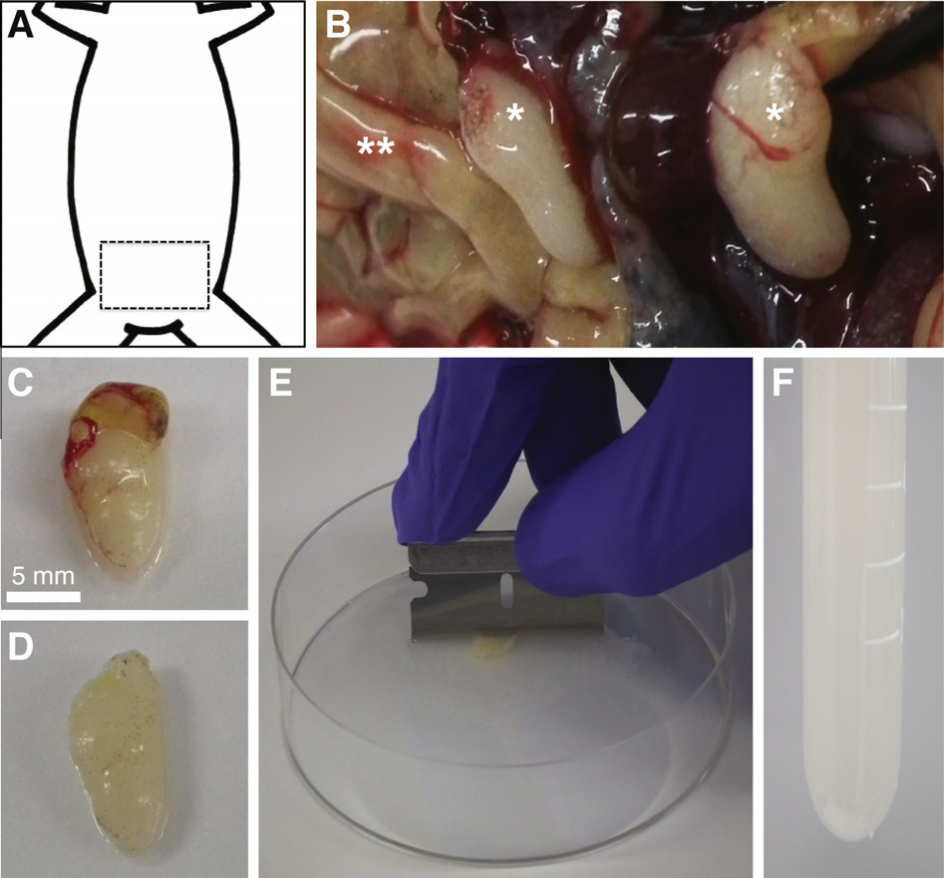
Preparation of demembranated *Xenopus* sperm nuclei. (A) Schematic representation of the body portion of a frog; dashed box indicates the expected location of the testes internally. (B) Testes, *in situ*, post-incision. ^∗^testes; ^∗∗^digestive system. (C and D) Excised testis, pre- (C) and post- (D) removal of extraneous tissue and blood vessels. (E) Chopping the testes in a plastic Petri dish using a razor blade. (F) Filtered sperm solution.

1. Place male frogs in MS222 solution in individual opaque chambers. Batches of 3–4 frogs can conveniently be processed together. When their heads drop under water and they stop moving when touched, check for life by placing a forefinger deep into the frog’s mouth – if the frog gives a reflex choking response then it is alive. Keep the frog in MS222, checking every minute or so until the reflex is lost. At this point remove the frog from the MS222 and place on its back on a protected work surface. Ensure death by quickly opening up the abdomen using sharp scissors or a scalpel and snipping the main arteries around the heart.
2. As soon as possible after this remove the testes, cutting carefully so as not to cut or damage them, and place in EB solution on ice. The testes are located in the lower abdominal region (Fig. 3A); they are most easily accessible when the frog is placed on its back and entered ventrally. Often partially hidden in the body cavity they are readily distinguishable morphologically from the similarly coloured digestive system (Fig. 3B); the testes are approximately bean/egg shaped and ivory in colour and are typically 0.75–1.5 cm in length (Fig. 3C). Although the vast majority of animals are found with two testes, specimens with only one, or more rarely three, have been seen; within one animal, the testes may not be of equal size.
3. Wash testes in ∼20 ml of EB in a 9 cm Petri dish. Carefully clean the testes with 2 pairs of dissection forceps to remove any blood vessels and extraneous tissue, being careful not to burst the testes (Fig. 3D).
4. Transfer the cleaned testes to a clean Petri dish containing 10 ml EB. Hold down a testis with the dissection forceps whilst using a razor blade to chop the testis as finely as possible (Fig. 3E). Pool all chopped material and store on ice.
5. Filter the homogenate through a 25 μm mesh nylon filter (e.g. Nitex or Nybolt) mounted over the end of a 50 ml tube. This can be done conveniently by cutting a large square from the top of a screw cap lid. Use the cut lid to secure a square of nylon mesh over the end of the tube containing the sperm; the contents of the tube can then be poured out into another container through the filter mounted in the lid. The filtered material should look quite cloudy (Fig. 3F).
6. Transfer the filtered material to a 15 ml tube and spin at 2000*g*, in a swinging bucket rotor for 5 min at 4 °C. If the supernatant appears cloudy after this spin, remove the supernatant and re-spin. Pool the pelleted fractions. If the sperm preparation contains significant contamination of erythrocytes these will appear as a red fraction at the bottom of the pellet. These cells can be separated by careful resuspension and transfer of the sperm to a second tube. The recovered sperm should be respun and the still pelleted bloods cells in the original tube discarded.
7. Resuspend the pellet in a total volume of 0.5 ml SuNaSp at room temperature for each testis. Then supplement with 25 μl lysolecithin for each testis and incubate for 5 min at room temperature. After 5 min check for demembranation of the sperm by mixing 1 μl of sample with 1 μl of Hoechst 33258 (20 μg/ml) and view by UV microscopy. Demembranated sperm appear as small fat “squiggles” which stain bright blue with Hoechst; non-demembranated sperm will not stain with Hoechst. If <95% are demembranated, then repeat lysolecithin treatment after respinning and resuspending the sperm in fresh SuNaSp.
8. Centrifuge the demembranated sperm at 2000*g* in a swinging bucket rotor for 5 min at 4 °C. Quench the lysolecithin by resuspending the total pelleted material in 0.5 ml SuNaSp containing 3% BSA for each testis.
9. Centrifuge again and resuspend the pellet in 0.5 ml EB for each testis. Repeat. Resuspend the washed pellet in 100 μl EB + 30% glycerol for each testis used.
10. To count the sperm, make a small aliquot of the resuspended pellet and dilute 1:100 in EB. Use a haemocytometer to count the number of sperm and large somatic-type nuclei. The sperm may look different depending on the angle they are viewed from, ranging from a squiggle (side view) to a circle (top view). Preparations typically contain 1–5% somatic nuclei. The count is repeated 4 times to ensure statistical accuracy. The mean number is then calculated, with somatic nuclei given a double weighting (because they are diploid). The DNA concentration of the preparation can be determined, given that the *Xenopus* haploid genome = 3 pg DNA. We typically recover 100–200 μg DNA (33,000,000–66,000,000 haploid nuclei) per testis.
11. Dilute the stock, in EB plus 30% glycerol, to give a final concentration of 400 ng DNA/μl (133,000 haploid nuclei/μl). Freeze in 80 μl aliquots and store in Eppendorf tubes at −80 °C.

## 3 Use of *Xenopus* egg extracts

To ensure the integrity and determine the cell cycle status of the final extract we assess both nuclear assembly (Section 3.1) and replication kinetics (Section 3.2) upon addition of sperm nuclei to the extracts plus and minus Ca^2+^. To test for the late onset of apoptosis we follow nuclei morphology over the course of 8 h, during which time the nuclei should remain stable. Extracts may escape from metaphase arrest during preparation and form nuclei in the absence of exogenous calcium ions but otherwise remain stable.

In order to use the extract, the required number of frozen beads are transferred to an Eppendorf tube and thawed in a room-temperature water bath. The extract should be used immediately after thawing and should be kept at 4 °C or on ice during processing until the replication reaction is started. DNA replication demands a maintained energy supply. In order to keep ATP levels high throughout the duration of the reaction the extract is supplemented with a source of high-energy phosphate [5]. The addition of an energy regenerator (ER) solution (25 mM phosphocreatine and 15 μg/ml creatine phosphokinase at final concentration) to freshly thawed extract, immediately before use, provides the required activity. Frozen extracts do not efficiently support protein synthesis and so do not usually progress into first mitosis; in order to ensure this cycloheximide can be added at 250 μg/ml. Metaphase arrested extracts can be activated to progress into anaphase and the first mitotic cell cycle upon addition of 0.3 mM CaCl_2_, mimicking the calcium wave that is induced on fertilization [2]. We usually activate the extract for 15 min at 23 °C before template DNA addition. The capacity of the extract to assemble demembranated sperm into nuclei is ∼30 ng DNA/μl (10,000 haploid nuclei per μl), close to the concentration of chromosomal DNA in embryos at the midblastula transition [5]. With sperm nuclei, we typically use final DNA concentrations of 3–10 ng DNA/μl extract. The extract has a similar capacity to replicate pre-formed interphase nuclei [29]. Dilution of the extract severely compromises nuclear assembly, so the total volume of additions should be kept to <20% extract volume. *In vitro* replication reactions are performed at 23 °C.

### 3.1 Nuclear assembly

**Fig. 4:**
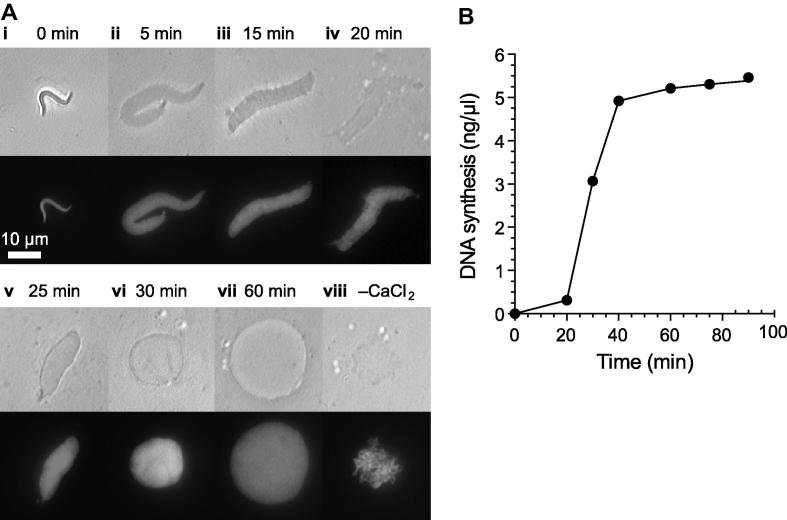
Nuclear assembly and DNA replication in *Xenopus* egg extracts. (A) Timecourse of nuclear formation in *Xenopus* egg extracts. Sperm nuclei were incubated in metaphase arrested *Xenopus* egg extract released into interphase by addition of 0.3 mM CaCl_2_. Nuclear formation was followed over the course of 60 min by phase contrast (upper panels) and UV (lower panels) microscopy (i–vii). Sperm nuclei incubated in extract in the absence of CaCl_2_ were visualised after 60 min (viii). Bar = 10 μm. (B) The replication kinetics of sperm nuclei added to interphase egg extract as determined by the ‘TCA replication assay’.

The replication of sperm nuclei in egg extract is dependent upon nuclear assembly and each nucleus forms a discrete unit of replication [30]. Both the time of nuclear assembly and the length of S phase vary from extract to extract. Typically nuclei assemble in extract between 20 and 40 min after sperm addition with DNA replicating completely within 20–40 min after that. The kinetics of DNA replication in a sample is absolutely dependent on the efficiency of nuclear assembly and at increased concentrations of DNA, or in diluted extracts e.g., upon immunodepletion, assembly is less efficient resulting in slower DNA replication. In typical reactions, with DNA concentrations of 3–10 ng/μl, sperm form nuclei and enter S phase ∼30 min after addition to extract and complete replication within 1 h (Fig. 4). Since the initiation of DNA replication in whole egg extract is dependent on nuclear assembly it is crucial that this is monitored in every experiment to rule out the non-specific inhibition of DNA replication.

The extent of nuclear assembly can be followed under the microscope (Fig. 4A): mix 1 μl extract sample with 1 μl Hoechst 33258 (20 μg/ml) and view the assembling nuclei by UV and phase contrast light microscopy. Within 5 min of addition to extract, the sperm nuclei undergo nucleoplasmin-mediated decondensation and increase in size (compare Fig. 4A-i and -ii). Over the next 15–25 min the decondensed chromatin acquires nuclear membrane components and these can be visualized by phase contrast as black spots on the surface of the sperm (Fig. 4A-iii and -iv). Only once the nuclear membrane has formed and nuclear assembly is complete can a solid black line be seen around the nuclei (Fig. 4A-v and -vi). The nuclei may continue to grow becoming ever larger in time (Fig. 4A-vii). The shape of nuclei formed in individual extracts varies. In our experience we find that nuclei formed in metaphase arrested extracts activated *in vitro* appear larger and typically more circular than those formed in extracts prepared from activated eggs or in those extracts that escaped metaphase arrest during preparation.

In the absence of CaCl_2_ addition sperm added to a metaphase arrested extract will not form nuclei. After primary nucleoplasmin mediated decondensation chromatin in metaphase arrested extract is seen to recondense and form into a network of condensed rod like chromosomes that separate and resolve in time (Fig. 4A-viii).

### 3.2 TCA replication assay

*Xenopus* egg extracts will incorporate a range of modified dNTPs into nascent DNA [5,30]. To accurately quantify the replication kinetics of DNA added to the extract an assay based on the incorporation of radiolabelled nucleotide into replicating DNA is used [5,23]. Briefly, egg extracts, supplemented with energy regenerator, cycloheximide and CaCl_2_ to which sperm nuclei or another DNA source are added, are incubated as required with α^32^P-dATP. Reactions, terminated by addition of a solution containing SDS and protease, are then added to cold 10% TCA to facilitate precipitation of the DNA. The precipitated DNA is separated from the unincorporated radiolabel by filtration and the level of radioactivity in each sample is measured by scintillation counting; the extent of DNA replication is determined by calculating the percentage of incorporated radiolabel. A typical time course is shown in Fig. 4B.

#### 3.2.1 Materials and reagents

*Egg extract and demembranated sperm nuclei at a known concentration*

*Energy regenerator (ER) (FS to 4* *°C):* 1 M phosphocreatine K salt, 600 μg/ml creatine phosphokinase in 10 mM HEPES–KOH pH 7.6.

*Cycloheximide (FS to 4 °C):* 10 mg/ml in H_2_O.

*CaCl_2_ (FS to 4 °C):* 50 mM in H_2_O.

*Stop-C:* 0.5% w:v SDS, 5 mM EGTA, 20 mM Tris HCl, pH 7.5.

*Proteinase K:* 20 mg/ml proteinase K, 50% v:v glycerol, 10 mM Tris HCl, pH 7.5.

*5% TCA (stored at 4 °C):* 5% w:v TCA, 0.5% w:v Na_4_P_2_O_7_·10H_2_O.

*10% TCA (stored at 4 °C):* 5% w:v TCA, 2% w:v Na_4_P_2_O_7_·10H_2_O.

α^32^P-dATP (high activity: typically 10 mCi/ml).

25 mm Paper filter discs.

25 mm Glass microfibre filter discs.

Vacuum manifold (Millipore).

#### 3.2.2 Replication assay procedure

1. Supplement egg extract with energy regenerator (ER; 1/40 stock), cycloheximide (1/40 stock; 250 μg/ml final concentration), CaCl_2_ (1/167 stock; 0.3 mM final concentration) and sperm nuclei at the required concentration, with 50 nCi/μl α^32^P-dATP and incubate at 23 °C.
  Individual samples need be no more than 10–20 μl in volume. If multiple samples are to be prepared from one extract treatment, for example when performing a timecourse to determine replication kinetics, aliquots should be made as early as is appropriate; aliquots of 3–5 μl are sufficient for this purpose.
2. Terminate the reaction by addition of 160 μl Stop-C + freshly added 0.2 mg/ml proteinase K, mix well and incubate at 37 °C for 30 min.
3. Precipitate the digested sample with 4 ml of 4 °C 10% TCA for at least 30 min at 4 °C.
4. Spot 40 μl of the TCA sample onto a paper filter disc for measurement of total ^32^P.
5. Filter the remainder of the sample under vacuum on a manifold through a glass fibre filter.
6. Wash the glass fibre filter twice with 8 ml of 4 °C 5% TCA and then with 8 ml of ethanol.
7. Insert each filter into a scintillation counter tube, and add enough scintillant (e.g. Ultima Gold F; Perkin Elmer) to entirely wet the filter. Measure the ^32^P on the filters in a scintillation counter; a wide open or ‘Cerenkov’ channel is appropriate because the filter reduces the recorded energy of emission.

The extract contains endogenous dNTP pools of ∼50 μM [5]. Dividing the ^32^P incorporated into DNA captured on the glass fibre filter by the total ^32^P on the paper filter gives the percentage of total ^32^P incorporated into DNA. The total amount of DNA synthesized, expressed in ng DNA/μl extract, can then be calculated by multiplying percent total ^32^P incorporated by a factor of 0.654 [5]. This calculation assumes an average molecular weight of dNMPs of 327 Da and equal quantities of all four dNTPs incorporated into DNA (weight of dNMP incorporated in ng/μl = percent total ^32^P incorporated/100 × 50 × 10^−6^ × 4 × 327 × 10^3^).

## 4 Isolation of chromatin and nuclei assembled in egg extract

Because *X. laevis* egg extract is a cell-free system, the process of chromatin or nuclear isolation is relatively simple. It therefore provides a considerable advantage over cell based model systems when considering the study of chromatin associated proteins.

In brief, extract with replicating nuclei at a chosen stage of DNA replication is diluted with isolation buffer and then spun through a sucrose cushion. After the spin, chromatin particles or nuclei accumulate at the bottom of the tube as they are sufficiently dense to sediment through the concentrated sucrose solution while the vast majority of extract proteins remain above the cushion.

### 4.1 Isolation of chromatin for analysis by immunoblotting

As isolation of chromatin from the extract is comparatively simple it can be easily performed as a timecourse experiment to assess the dynamic timing of protein association. Additionally the effect of various inhibitors and drug treatments can be analysed in parallel.

Because of the time required to process samples, taking multiple timepoints less than 10 min apart requires considerable experience. To make sure that each sample has been isolated with comparable efficiency (as chromatin pellets can be lost or contaminated with the extract) it is very important to have good loading controls. Histone proteins are very abundant on chromatin while having a very small molecular weight, allowing them to be visualised by either the Coomassie staining [31] or immunoblotting [32] of the lower portion of a protein gel (e.g. pre-cast 4–12% gradient gels; Invitrogen). In this way they can provide a useful loading control for the remaining (upper) portion of the gel which is immunoblotted for any proteins of interest. It should be noted that the histone content will approximately double as all the template DNA is replicated during S phase.

Another important issue to consider is the specificity of a protein’s association with isolated chromatin. Some proteins can precipitate in the isolation buffer in the absence of DNA and so their sedimentation through the sucrose cushion may not be due to their association with chromatin. To determine whether this is the case for your protein of interest it is necessary to perform a control mock ‘chromatin isolation’ of extract without addition of sperm for each treatment tested.

#### 4.1.1 Materials and reagents

*Egg extract and demembranated sperm nuclei at a known concentration*

*Energy regenerator (ER):* 1 M phosphocreatine and 600 μg/ml creatine phosphokinase in 10 mM HEPES–KOH pH 7.6.

*Cycloheximide:* 10 mg/ml in H_2_O.

*CaCl_2_:* 50 mM in H_2_O.

*Nuclear isolation buffer (NIB):* 50 mM HEPES–KOH pH 7.6, 50 mM KCl, 5 mM MgCl_2_, 0.5 mM spermidine, 0.15 mM spermine, 0.1% Triton X-100, 2 mM DTT, 1 μg/ml leupeptin, 1 μg/ml pepstatin, 1 μg/ml aprotinin, 0.1 mM PMSF added fresh. Optional NIB additions: 2.5 mM Mg-ATP (from stock of 250 mM ATP, 250 mM MgCl_2_ pH 6.7 with NaOH), phosphatase inhibitors: 25 mM Na glycerophosphate, 0.1 mM Na_3_VO_4_, 0.1 μM microcystin-LR [23].

NIB + 15% or 30% sucrose.

23 °C incubator.

Colourless 1.5 ml Eppendorf tubes transparent at the tip, avoid low retention tubes.

Cooled microfuge with a swinging-bucket rotor.

Microfuge with a fixed-angle rotor.

SDS or LDS gel loading buffer.

Nu-PAGE 4–12% gradient pre-cast gels, Nu-PAGE MOPS running buffer (Invitrogen).

Pre-stained protein marker such as PageRuler™ Prestained Protein Ladder (Thermo Scientific).

Coomassie stain (0.1% Coomassie Brilliant Blue R-250, 40% methanol, 10% acetic acid) and destaining solution (40% methanol, 10% acetic acid).

#### 4.1.2 Chromatin isolation procedure

**Fig. 5:**
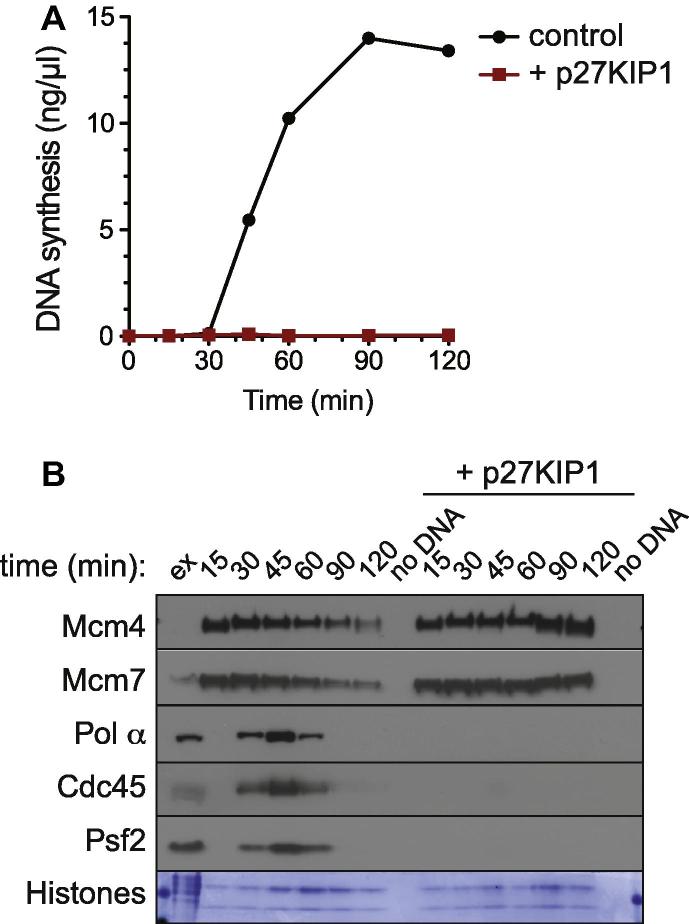
Timecourse of replication factors association with chromatin. The replication reaction was assembled plus or minus 100 nM CDK inhibitor p27KIP1. (A) DNA synthesis was assayed by α^32^P-dATP incorporation. (B) Chromatin was isolated at the indicated times, separated by SDS–PAGE and immunoblotted with antibodies against the indicated replication proteins. The lower portion of the protein gel was stained with Coomassie for histones.

1. Set up the appropriate reaction conditions, as described in Section 3. In order to maximise the protein signal for immunoblotting, template DNA concentrations can be increased to a maximum of no more than 20 ng/μl. It should be noted that at increased DNA concentrations replication kinetics may be altered. For each chromatin sample, we typically use 10–20 μl extract. Aliquot out all timepoints or treated samples into different tubes as early as is appropriate and incubate at 23 °C for the required length of time. A replication assay run in parallel serves as an important control for chromatin isolation. An example experiment is shown in Fig. 5.
2. To stop the reaction, add 0.5 ml of ice cold NIB buffer to the extract and mix thoroughly but gently. Diluting the extract is important for two reasons: firstly it reduces the density of the neat extract which is necessary to allow the chromatin to be pelleted, and secondly it decreases the viscosity of the extract, allowing more rapid chromatin isolation. Underlay the diluted extract slowly with 100 μl of NIB + sucrose to create a cushion. We most frequently use NIB + 15% sucrose for chromatin isolation but in cases where greater stringency is required NIB + 30% sucrose can be used.
3. Centrifuge extract plus cushion at 2100*g*, at 4 °C, for 5 min in a swinging-bucket rotor. Tubes must be handled carefully to avoid mixing the layers.
4. After centrifugation, remove the buffer from above the cushion. In order to reduce contamination of the chromatin sample with diluted extract, add a further 100–200 μl NIB on top of the cushion and then remove it again. Repeat this cushion wash two more times. Then carefully remove most of the cushion solution – leaving about 15 μl behind, taking care not to aspirate the chromatin pellet which is usually not visible at this stage but forms an indistinct mass at the bottom of the tube.
5. To facilitate isolation of the chromatin mass, centrifuge the tubes at top speed in a microfuge (fixed angle, >10,000*g*) for 2 min to focus the pellet. If the tubes are placed in the rotor in a defined orientation, this helps determine where in the tube the chromatin pellet will be located. Carefully remove all the remaining liquid from the tube using a fine pipette tip. At this point the chromatin pellet should be visible as a tiny opaque circular pellet on the side of the tube.
6. Add 20 μl SDS loading buffer to each tube, boil and separate by SDS–PAGE. In order for histones to be quantified, do not run the dye front off the bottom of the gel.
7. The lowest portion of the gel can be cut off and stained with fresh Coomassie solution to visualise histones [31] (Fig. 5B, lowest panel). The remainder of the gel can be transferred onto a PVDF membrane and analysed by immunoblotting (Fig. 5B, upper panels). Alternatively, histone abundance can be assessed by immunoblotting using anti-histone H3 or H4 antibodies (rabbit polyclonal; Upstate Biotechnology) [32].

### 4.2 Release of native chromatin proteins into solution

In order to analyse native chromatin-bound proteins and protein complexes using techniques such as gel filtration or immunoprecipitation, these proteins have to be stripped off the DNA in the gentlest possible way. To achieve this chromatin can be pelleted through a sucrose cushion under gentle buffer conditions and then DNase used to fully digest isolated DNA. Phosphatase inhibitors can be included in the isolation buffer to preserve protein phosphorylation status, which may be crucial for the stability of some complexes. In these experiments the salt concentration used in the isolation buffer needs to be established for the particular protein studied: some protein complexes require a higher salt concentration in order to become soluble while others can fall apart when subjected to more salt [13]. As a compromise our starting point is 100 mM KOAc.

#### 4.2.1 Materials and reagents

*Assembled replication reaction*

*Acetate nuclear isolation buffer (ANIB):* 50 mM HEPES–KOH pH 7.6, 100 mM KOAc, 10 mM MgOAc, 0.1% Triton X-100, 2.5 mM MgATP (from stock of 250 mM ATP, 250 mM MgCl_2_ pH 6.7 with NaOH), 0.5 mM spermidine, 0.15 mM spermine, 25 mM Na glycerophosphate pH 7.6, 0.1 mM Na_3_VO_4_, 0.1 μM microcystin-LR [22], 1 μg/ml leupeptin, 1 μg/ml pepstatin, 1 μg/ml aprotinin, 0.1 mM PMSF and 1× Halt protease inhibitor cocktail (Thermo Scientific) both added freshly.

ANIB + 30% sucrose.

15 or 50 ml tubes.

1.5 ml Eppendorf tubes.

Plastic pipette/droppers with long neck and fine tip.

Cooled centrifuge with a swinging-bucket rotor able to spin at 2500*g.*

Cooled microfuge with a fixed-angle rotor.

Benzonase (Novagen).

Optionally – water bath sonicator (Bioruptor, Diagenode).

#### 4.2.2 Chromatin protein isolation and release procedure

**Fig. 6:**
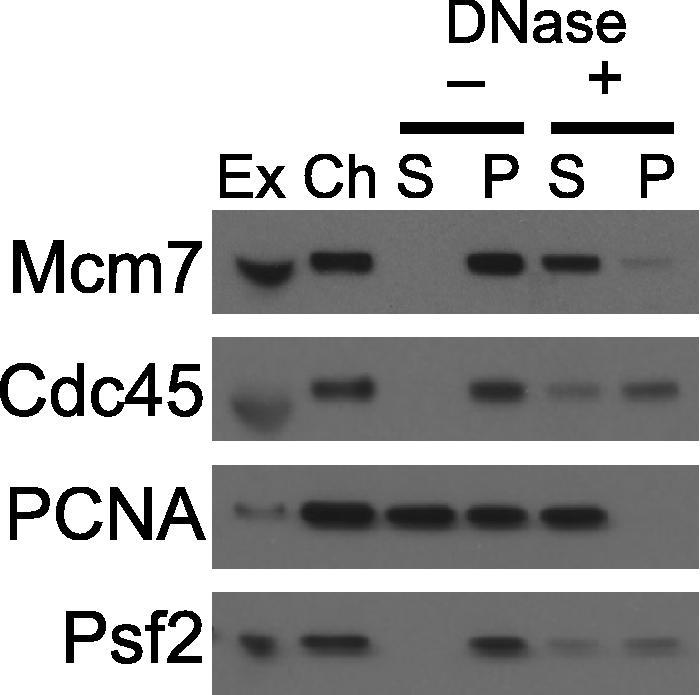
Release of native proteins from chromatin. Chromatin was isolated from egg extract in the middle of S-phase (when replisome proteins peak on chromatin) and proteins were optionally released from chromatin with benzonase. (Ex), 0.5 μl egg extract; (Ch), isolated chromatin, from 5 μl extract, after first centrifugation; other lanes correspond to insoluble pellet (P) and soluble supernatant (S) from 5 μl extract after a second centrifugation. Samples were separated by SDS–PAGE and immunoblotted with antibodies against indicated proteins.

1. Set up reactions as described in Section 3 and incubate at 23 °C. If downstream fractionation (such as gel filtration or immunoprecipitation) is to be performed larger amounts of chromatin are required than for simple immunoblotting analysis (Section 4.1) and extract volumes of >500 μl are typically used.
2. At the end of the incubation stop the replication reaction by diluting the extract with 10 volumes of ice cold ANIB. Mix well by inverting the tube several times. Underlay with ANIB + 30% sucrose using a plastic fine tip pipette/dropper (about 1 ml in a 15 ml tube and 2.5 ml in a 50 ml tube).
3. Centrifuge at 2500*g* for 10 min at 4 °C in a swinging bucket rotor. If after the spin there are white threads of chromatin visible floating throughout the sucrose cushion repeat the spin.
4. Aspirate the buffer from above the cushion. To wash soluble protein from the top of the cushion, add a volume of ANIB buffer to the top of the cushion, and then aspirate it. The volume of ANIB buffer added should be equal to the volume of the cushion. Repeat the cushion wash procedure a total of three times.
5. At this stage the chromatin pellet forms a white fluffy mass. Remove as much of the cushion solution from above the pellet as possible and transfer the pellet in the remaining solution to a 1.5 ml tube using a tip with the end cut off. Add fresh ANIB + 30% sucrose to a final volume ¼ that of the original extract sample (use this additional buffer to wash the original tube and transfer any chromatin remaining).
6. Resuspend the chromatin pellet by pipetting up and down until the chromatin mass is broken up and no more chromatin threads are visible. Take a 5–10 μl aliquot and add SDS loading buffer to provide a ‘whole chromatin’ sample for checking the quality of preparation and the efficiency of chromatin resolubilisation.
7. Remove 5–10 μl of resuspended chromatin into a new tube (for the ‘no DNAse’ sample) and process it alongside the main sample. To the rest of resuspended chromatin add Benzonase up to concentration of 2 U/μl. Incubate for 10 min at room temperature. After Benzonase treatment no chromatin particles should be visible in the sample.
8. Optional: sonicate the chromatin sample, for example using a Bioruptor (Diagenode) for 5 min at a medium power setting with 10 s of sonication and 45 s breaks. This ensures that all fragments of chromatin are broken into pieces and accessible to Benzonase. Sonication made no significant difference for the release efficiency of the proteins we have studied so far but may be important for others.
9. Spin both samples (main and ‘no DNAse’) for 5 min at 20,000*g* at 4 °C in a fixed-angle rotor. Remove the supernatant promptly to new tubes. This soluble chromatin sample can be used for further analysis, such as gel filtration or immunoprecipitation.
10. Take a 5–10 μl aliquot from the supernatant samples for immunoblotting and add SDS loading buffer.
11. Resuspend the remaining pellets in ANIB to a volume equal to that of the removed supernatant, take a 5–10 μl aliquot and add SDS loading buffer.
12. Analyse test samples (‘whole chromatin’, ‘main supernatant’, ‘main pellet’, ‘no DNase supernatant’, ‘no DNAse pellet’, plus 0.5 μl whole extract) by SDS–PAGE and immunoblot for proteins of interest (example shown in Fig. 6). The whole chromatin sample shows how much of the protein was bound to chromatin in the first place (Fig. 6, ‘Ch’). The presence of proteins in the supernatant without digestion (Fig. 6, −DNase S) shows contamination of the chromatin sample with extract or weakly-associating chromatin proteins. For example, Mcm2-7 proteins are tightly bound to chromatin prior to S phase and should not be released at this point [13]. The abundance of proteins in the supernatant after digestion (Fig. 6, +DNase S) in comparison to what is left in the pellet (Fig. 6, +DNase P) indicates the efficiency of protein release – for Mcm2-7 this should be approximately 80–100% in 100 mM KOAc.

As noted above, some proteins may require relatively higher salt concentrations to become soluble after digestion of chromatin. However, isolation of chromatin in high salt buffer can result in proteins being stripped off sedimenting chromatin and thus becoming lost during preparation. To solve this problem, we isolate chromatin in 50–100 mM KOAc ANIB buffer and increase salt concentration only after digestion of chromatin with Benzonase. We add 2 M KOAc stock solution to adjust the salt concentration and incubate the samples for an additional 5 min at room temperature before spinning in a fixed-angle rotor. As above, a 5–10 μl aliquot can be taken out before salt adjustment and analysed by SDS–PAGE alongside other test samples to determine the effect of increased salt concentration on the recovery of proteins.

### 4.3 Isolation of nuclei for nuclear transfer

Intact nuclei can be isolated from *Xenopus* extract in order to analyse them physiologically, for example to determine different stages in the initiation of DNA replication [8,17,32]. Nuclei can be isolated from extracts supplemented with a specific replication inhibitor, or from which a specific protein has been immunodepleted and then added to a second extract to assess the process of release from inhibition or to determine protein function; any number of combinations of protein depletion or drug addition to the first or second extract can be envisaged, depending on the question of interest. Isolation of intact nuclei assembled in egg extract is in principle similar to chromatin isolation (Section 4.1) but it is crucial to isolate nuclei without any detergent to keep the nuclear envelope in place. In addition, using a double cushion (15% sucrose and 30% glycerol) prevents the nuclei from being spun into a compact mass attached to the bottom of the tube that prevents their easy resuspension. It should be noted that nuclear envelopes assembled *in vitro* are very leaky once out of neat extract, and the majority of soluble nucleoplasmic protein is lost once nuclei are exposed to buffer [17]. To efficiently retain nucleoplasmic protein, nuclei must be ‘floated’ in neat extract [22].

#### 4.3.1 Materials and reagents

*Buffer A:* 15 mM Tris–HCl pH 7.4, 60 mM KCl, 15 mM NaCl, 2 mM DTT, 0.5 mM spermidine, 0.15 mM spermine, 1 μg/ml leupeptin, 1 μg/ml pepstatin, 1 μg/ml aprotinin, 0.1 mM PMSF).

Buffer A + 15% sucrose.

Buffer A + 30% glycerol.

Cut 200 μl tips (the very end of the tip is cut off to avoid damaging nuclei while resuspending).

Colourless 1.5 ml Eppendorf tubes transparent at the tip, avoid low retention tubes.

Cooled microfuge with a swinging-bucket rotor.

#### 4.3.2 Nuclei isolation procedure

**Fig. 7:**
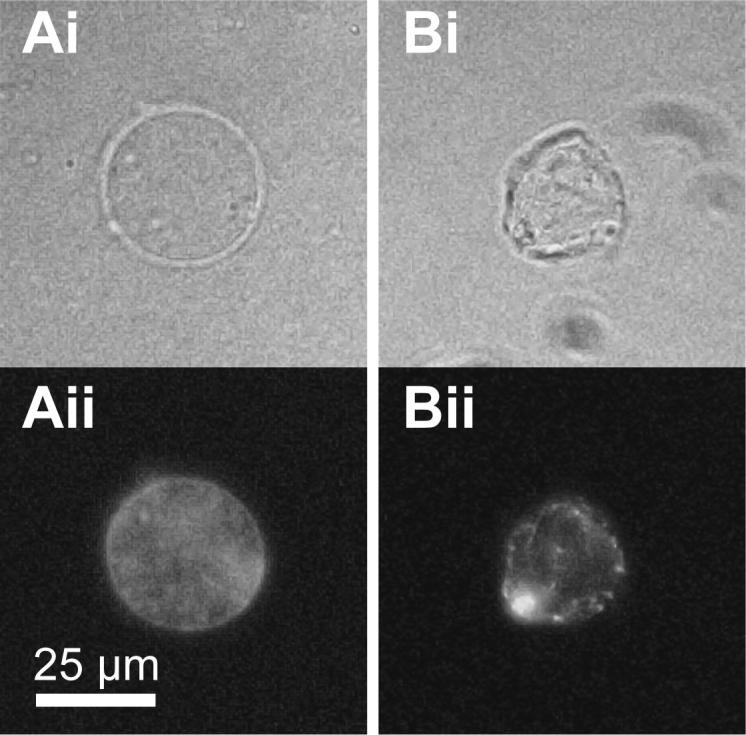
Isolated intact nuclei. Nuclei were isolated from egg extract 40 min after replication reaction assembly. (A) Nucleus before isolation. (B) Isolated nucleus. (i) Phase contrast light microscopy; (ii) DNA was stained with Hoechst 33258 and visualised by UV fluorescence microscopy.

1. Set up appropriate reactions as described in Section 3 and incubate at 23 °C. For this procedure we typically use 10–40 μl extract with template DNA concentrations of 3–20 ng/μl. It should be noted that at increased DNA concentrations replication kinetics may be altered. This is especially so in immunodepleted extracts.
2. After the required time of incubation, mix extract gently with 10 volumes of ice cold Buffer A and underlay with 100 μl of Buffer A + 15% sucrose (w:v).
3. Underlay the sucrose cushion with 5 μl of Buffer A + 30% glycerol (v:v).
4. Centrifuge samples in a swinging-bucket rotor at 2100*g*, 4 °C, 5 min.
5. Remove buffer carefully from above the sucrose cushion. Then carefully remove the cushion layer down to about 10 μl above the glycerol layer – an identical tube with 15 μl of liquid in the bottom can be used as a reference.
6. Resuspend the isolated nuclei in the remaining solution using a 200 μl pipette tip cut to prevent sheer forces. Determine the final volume by setting the pipette to exactly take up the sample. If necessary supplement the samples with Buffer A + sucrose to bring them to the volume of the largest sample. Assuming equal recovery, calculate the concentration of isolated nuclei depending on the volume of original extract and concentration of sperm used. Add isolated nuclei to the second extract in the place of demembranated sperm. An example of nuclei before and after isolation is shown in Fig. 7.

## 5 Concluding remarks

In this article we have described the methods currently in use in our laboratory for the study of DNA replication using the *Xenopus* cell-free system. In addition to the protocols describing the preparation of egg extracts and demembranated sperm nuclei, we have also provided a comprehensive introduction to these methods, detailing important background information and highlighting critical steps in the procedure. We also describe a range of primary assays that can be performed using the extract that allow quantification of DNA replication and chromatin isolation. These recently developed and revised techniques provide a practical starting point for investigation of protein function using this system.
